# High Density Lipoprotein Structural Changes and Drug Response in Lipidomic Profiles following the Long-Term Fenofibrate Therapy in the FIELD Substudy

**DOI:** 10.1371/journal.pone.0023589

**Published:** 2011-08-24

**Authors:** Laxman Yetukuri, Ilkka Huopaniemi, Artturi Koivuniemi, Marianna Maranghi, Anne Hiukka, Heli Nygren, Samuel Kaski, Marja-Riitta Taskinen, Ilpo Vattulainen, Matti Jauhiainen, Matej Orešič

**Affiliations:** 1 Technical Research Centre of Finland, Espoo, Finland; 2 Aalto University School of Science, Department of Information and Computer Science, Helsinki Institute for Information Technology, Espoo, Finland; 3 Department of Internal Medicine and Medical Specialties, Sapienza University, Rome, Italy; 4 Division of Cardiology, Department of Medicine, University of Helsinki, Helsinki, Finland; 5 Department of Physics, Tampere University of Technology, Tampere, Finland; 6 Department of Applied Physics, Aalto University School of Science and Technology, Espoo, Finland; 7 MEMPHYS – Center for Biomembrane Physics, University of Southern Denmark, Odense, Denmark; 8 National Institute for Health and Welfare, Helsinki, Finland; 9 Institute for Molecular Medicine Finland, Helsinki, Finland; University of Maribor, Slovenia

## Abstract

In a recent FIELD study the fenofibrate therapy surprisingly failed to achieve significant benefit over placebo in the primary endpoint of coronary heart disease events. Increased levels of atherogenic homocysteine were observed in some patients assigned to fenofibrate therapy but the molecular mechanisms behind this are poorly understood. Herein we investigated HDL lipidomic profiles associated with fenofibrate treatment and the drug-induced Hcy levels in the FIELD substudy. We found that fenofibrate leads to complex HDL compositional changes including increased apoA-II, diminishment of lysophosphatidylcholines and increase of sphingomyelins. Ethanolamine plasmalogens were diminished only in a subgroup of fenofibrate-treated patients with elevated homocysteine levels. Finally we performed molecular dynamics simulations to qualitatively reconstitute HDL particles *in silico*. We found that increased number of apoA-II excludes neutral lipids from HDL surface and apoA-II is more deeply buried in the lipid matrix than apoA-I. In conclusion, a detailed molecular characterization of HDL may provide surrogates for predictors of drug response and thus help identify the patients who might benefit from fenofibrate treatment.

## Introduction

Cardiovascular disease (CVD) events are responsible for 75–80% of all mortalities in patients with diabetes [1]. Patients with type 2 diabetes commonly have atherogenic dyslipidaemia, characterized by abnormalities in lipids and apolipoproteins, e.g., elevated triglycerides (TGs), decreased high-density lipoprotein cholesterol (HDL-C), small dense low-density lipoprotein (LDL) particles and increased apoB-100 to apoA-I ratio. Despite the unequivocal success of statin therapy to reduce major cardiovascular events, considerable residual risk persists in people with type 2 diabetes [2], [3]. It is therefore necessary to target the major components, i.e., high TGs and low HDL cholesterol in the malignant diabetic dyslipidemia to further reduces CVD events.

Fibrates, peroxisome proliferator activated receptor α (PPARα) agonists, are lipid lowering drugs which are recommended for patients with atherogenic dyslipidemia [4], [5]. The fibrate therapy is a promising approach to reduce TGs, improve improve distribution of LDL subpopulations and raise HDL-C. The possibility of fibrate therapy to benefit patients with type 2 diabetes at high CVD risk was the rationale for the design of the largest population-based fenofibrate study, Fibrate Intervention and Event Lowering in Diabetes (FIELD) [6]. The FIELD study recruited 9,795 patients, aged 50–75 years, irrespective of their prior CVD history or gender. The patients were randomized for micronised fenofibrate (200 mg/day) or placebo treatment in a double-blinded design for a period of 5 years. Unexpectedly, the fenofibrate study failed to achieve significant benefit over placebo in the primary endpoint of non-fatal and fatal coronary heart disease events, even though fenofibrate treatment significantly reduced CVD events in patients with dyslipidemias. In the FIELD study fenofibrate therapy decreased TGs by 29%, increased apoA-II by 28% but unexpectedly changes in HDL-C and apoA-I were minor showing barely 2% and 4% increases of HDL-C and apoA-I as compared to placebo group [6]. In a FIELD substudy, fenofibrate therapy induced notable changes in the distribution of HDL subspecies [7]. Growing evidence suggests that atheroprotective nature of HDL subspecies depends on the particle composition and size: apoA-I may be more effective than apoA-II and the greater the size of HDL particle, the higher the atheroprotective nature [7], [8], [9], [10].

Of interest, a robust increase in homocysteine (Hcy) levels was observed in patients assigned to fenofibrate therapy [11]. In the same study, a direct relationship between Hcy and apoA-II changes was observed. Hcy is an atherogenic amino acid and its high levels in blood are linked to increased incidence of CVD events [12], [13]. The effect of fenofibrate treatment on homocysteine levels is even more severe as compared to other fibrates [14]. Fibrate-induced increase of Hcy has raised the question whether this may compromise some of the beneficial effects of fibrate therapy on cardiovascular outcomes.

Traditional methods of lipoprotein analysis, which have relied on analyses of total protein, phospholipids, free cholesterol (FCho), cholesterol ester (ChoE) and TG content [15], cannot fully explain dysilipidemia and related apolipoprotein changes. Recent advances in mass spectrometry (MS)-based analytical platforms and related bioinformatics approaches have permitted the study of lipids at the molecular level [16], [17], [18], [19]. Molecular lipids may serve as better markers for specific metabolic phenotypes such as insulin resistance as compared to the total lipid class concentrations [20].

Detailed studies involving lipid and apolipoprotein molecular level information can contribute to better understanding of complex assembly of lipoprotein particles such as HDL. For example, we characterized the HDL lipid compositional differences in subjects with low and high HDL-cholesterol [21]. Using the information of the lipid composition of HDL particles in the two groups, HDL particles were then reconstituted *in silico* by performing large scale molecular dynamics simulations. The data from these simulations such as changes in lipid composition also induce specific spatial distributions of lipids within the HDL particles that may have important implications for HDL function.

We hypothesized that lipidomic study of HDL particles derived from the FIELD substudy patients will help elucidate (1) the effect of fenofibrate therapy in patients matched for Hcy levels, (2) the effect of elevated Hcy response and (3) the association between the Hcy response and fenofibrate therapy. Such study requires application of statistical models that address the specific questions related to Hcy and fenofibrate effects. Moreover, since fenofibrate therapy also affects apolipoprotein (mainly apoA-II) content in HDL particle, it is important to study the apolipoproteins and their interactions with other molecular lipids in HDL particles to better understand the complex structure of HDL in the context of response to therapy.

Here we performed global lipidomic analysis of HDL fractions in three groups of subjects: (1) High Hcy group representing highest quartile levels of Hcy, (2) Low Hcy group representing lowest quartile levels of Hcy and (3) placebo group, matching for Hcy levels in Low Hcy group at the study close-out. We applied a multivariate multi-way model to identify the effects of fenofibrate treatment, Hcy level and the synergistic effect of fenofibrate and Hcy. Based on the observed HDL compositional lipid and apolipoprotein changes in different study groups, we then performed large-scale molecular dynamic simulations to reconstitute HDL particles *in silico*.

## Results

### Characteristics of the study subjects

Clinical and biochemical characteristics of the study groups are shown in Table 1. Baseline and the study close-out samples for each variable were examined in Low and High Hcy groups, whereas in the placebo group only the study close-out samples were examined.

**Table 1:** Clinical and biochemical characteristics of the study groups.

	Fenofibrate Low Hcy		Fenofibrate High Hcy		Placebo
	Baseline	Close out	Baseline	Close out	Close out
**Gender (M/F)**	14/3	-	10/6	-	13/1
**BMI (kg/m^2^)**	29.5 (5.8)	29.5 (4.2)	32.1 (5.1)	30.4 (5.4)	31.4 (5.9)
**Smokers**	3	4	4	3	1
**Hcy (µmol/L)**	9.9 (2.3)	13.2 (2)	13 (3)#	27.4 (6.5)*	13.3 (0.7)
**TC (mmol/L)**	5.2 (0.5)	4.6 (0.9)	5 (0.4)	4.2 (0.6)	4.9 (0.4)
**HDL-C (mmol/L)**	1.1 (0.2)	1.1 (0.3)	1.1 (0.2)	1 (0.2)	1.2 (0.3)
**Triglycerides (mmol/L)**	1.6 (0.7)	1.4 (0.8)	1.9 (0.7)	1.4 (0.6)	1.6 (0.6)
**ApoA-I (mg/dl)**	102 (13.5)	102.4 (15.5)	103 (13.3)	106 (22.6)	102 (22.3)
**ApoA-II (mg/dl)**	24.3 (3.5)	29.9 (6.5)[Table-fn nt104] **	25.5 (4.9)	31.5 (5.1)[Table-fn nt104] **	24.8 (5.2)
**ApoA-I/ApoA-II ratio**	4.2 (0.4)	3.4 (0.3)[Table-fn nt104] **	4.1 (0.6)	3.3 (0.5)[Table-fn nt104] **	4.1 (0.5)
**HDL Size(nm)**	9.1 (2.7)	9.0 (3.4)	9.1 (2.7)	9.0 (3.7)	9.0 (6.2)
**PLTP (nmol/ml/h)**	5700 (1649)	5985 (1917)	6018 (1675)	5902 (1890)	7334 (1683)
**PON-1 mass (µg/ml)**	16.5 (8.9)	11.5 (11.2)	16.3 (5.7)	18.5 (6)	24.9 (14.6)

Values are mean± SD;

# p = 0.003 High Hcy vs Low Hcy at baseline;

*p<0.001 High Hcy vs Low Hcy and placebo at close out;

**p<0.05 Low Hcy vs placebo;

**p<0.05 High Hcy vs placebo; BMI = Body Mass Index; Hcy = homocysteine.

The groups were well matched for lipid and apolipoprotein levels at baseline. At the study close-out, apoA-I, HDL-C and TG levels were similar among the three groups, whereas subjects in both fenofibrate groups had higher apoA-II levels and lower apoA-I/apoA-II ratio as compared to the placebo group. Interestingly, in both fenofibrate groups apoA-I levels were comparable at baseline and close-out. Plasma PLTP activity and serum PON-1 mass at baseline and close-out were similar in both fenofibrate groups. At the study close-out PLTP activity and PON-1 mass in the placebo group showed a trend to higher values as compared to both fenofibrate groups.

**Figure 1:**
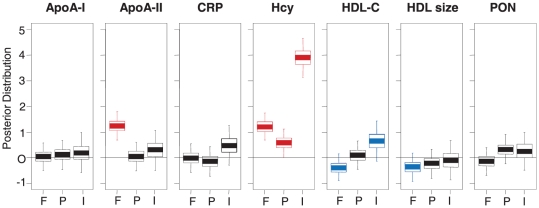
The effects of covariates on clinical variables. The effects fenofibrate (F), placebo (P) and interaction of fenofibrate and Hcy level (I) are shown by box-plots for each clinical variable. Positive fenofibrate effect indicates that both Low Hcy and High Hcy groups are upregulated by fenofibrate treatment. The combination of positive fenofibrate effect and positive interaction effect indicate that both Low Hcy and High Hcy groups are upregulated by fenofibrate treatment and for the High Hcy groups the effect is even stronger. The box plots show quartiles and 95% intervals of posterior mass of the effects; a posterior distribution above or below zero implies an effect is found. A positive effect (above zero) implies an upregulation, a negative effect (below zero) implies a down-regulation. Significant and almost significant effects are highlighted in red and blue, respectively.

We first applied multivariate multi-way modeling [22], [23] to identify the effects of covariates on clinical variables. The interaction effect indicates whether fenofibrate has a differential effect in Low and High Hcy groups. Our results confirmed the increased Hcy levels due to fenofibrate treatment (Figure 1) and the increase was even more pronounced for the High Hcy patients, indicated by the positive term corresponding to the interaction of fenofibrate and Hcy levels. Notably, among the HDL-associated apolipoproteins, apoA-II was upregulated by fenofibrate.

### Lipidomic analysis of HDL subfractions

**Figure 2:**
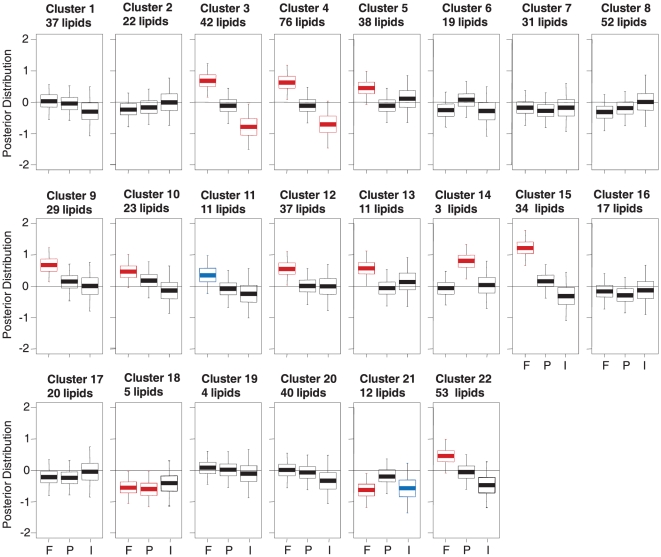
The effects of covariates on clusters of lipids. The effects of fenofibrate (F), placebo (P) and interaction of fenofibrate and Hcy level (I) are shown by box-plots for each cluster of lipids. The box plots show quartiles and 95% intervals of posterior mass of the effects; a posterior distribution above or below zero implies an effect is found. Positive fenofibrate effect indicates that both Low Hcy and High Hcy groups are upregulated by fenofibrate treatment. The combination of positive fenofibrate effect and negative interaction effect indicate that only the Low Hcy group is upregulated by fenofibrate treatment.

Molecular lipid profiles of HDL particles derived from blood samples collected at baseline and close-out were investigated by using ultra performance liquid chromatography coupled to mass spectrometry (UPLC-MS). A total of 615 lipid peaks were detected, of which 249 were identified. Lipidomics data analysis was performed using the multivariate multi-way analysis [22], [23]. In order to reduce the number of variables as part of the modeling, the method clustered the lipidomics data, resulting in 22 lipid clusters (Table 2). The effects of covariates on the lipid clusters are shown in Figure 2.

**Table 2:** Description of lipid clusters obtained from multivariate multi-way analysis.

Cluster	# of lipids*	Summary	Example of representative lipids
C1	37	HDL core lipids, mainly energy storage and dietary fat transporter lipids	TG(54∶3) and ChoE (18∶2)
C2	22	Mainly energy storage lipids	TG(50∶5) and TG(56∶9)
C3	42	Mainly abundant membrane and ether lipids	PC(36∶5) and PC(34∶4e)
C4	76	Ether-linked and energy storage lipids	PE(P-16∶0/20∶5) and TG(50∶2)
C5	38	Mainly abundant membrane lipids	PC(36∶6) and PC (34∶3)
C6	19	Mainly abundant membrane lipids	PC(38∶7) and PC(38∶6)
C7	31	Energy storage and dietary fat transporter lipids	TG(50∶4) and TG(56∶6)
C8	52	Energy storage and dietary fat transporter lipids	TG(48∶3) and TG(51∶1)
C9	29	Signal transduction and membrane lipids	SM(d18∶1/22∶0) and SM(d18∶1/24∶0)
C10	23	Membrane and ether lipids	PE(38∶4), PC(36∶5) and PE(P-18∶0/20∶5)
C11	11	Membrane lipids and energy storage lipids	PC(40∶2) and TG(48∶4)
C12	37	Mainly abundant phospholipids including ether-linked ones, as well as dietary fat storage lipids	PE(P-18∶0/22∶6), PC(36∶4e) and TG(53∶8)
C13	11	Membrane lipids	PE(36∶4)
C14	3	Not identified	-
C15	34	Mainly signal transduction and membrane lipids	SM(d18∶1/16∶1) and SM(d18∶1/18∶0)
C16	17	Energy storage and dietary fat transporter lipids	TG(58∶9)
C17	20	Mainly phospholipids, and certain core lipid	PC(36∶5) and ChoE(20∶5)
C18	5	Not identified	-
C19	4	Not identified	-
C20	40	Membrane lipids, ether-linked ethanolamine plasmalogens and HDL core lipids	PC(38∶6) , PE(P-16∶0/22∶6) and ChoE(22∶6)
C21	12	Abundant and anti-inflammatory lipids	LysoPC(22∶0) and LysoPC(16∶1)
C22	53	membrane and HDL core lipids	PC(40∶8) and ChoE(18∶1)

*Including both identified and unidentified lipid peaks.

Fenofibrate treatment induced specific changes in multiple lipid clusters. The main findings are summarized below:

- Sphingomyelin (SM)-rich clusters C9 and C15 were upregulated due to fenofibrate effect.
- Phosphatidylcholine (PC) rich clusters C5 and C22 as well as C10, C12 and C13 (mainly membrane and ether-linked lipids) were upregulated by fenofibrate.
- Clusters C3 and C4, both including ether phospholipids, were upregulated in the Low Hcy group due to fenofibrate effect; this is indicated by the combination of a positive fenofibrate effect and a negative interaction of fenofibrate and Hcy level.
- LysoPCs-specific cluster (C21) was down-regulated by fenofibrate effect.
- Cluster C18 with 5 unidentified lipids was down-regulated similarly with each treatment.
- Three unidentified lipids were upregulated in C14 of the placebo group.

**Figure 3:**
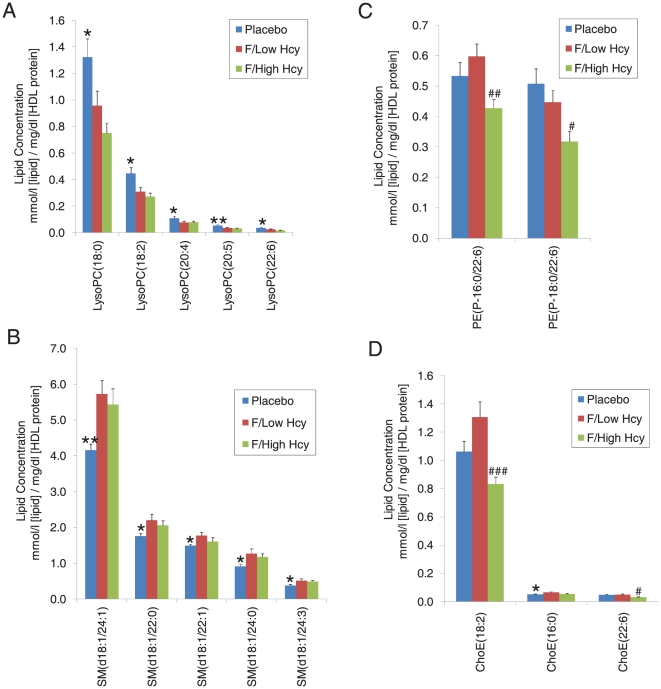
The concentrations of individual lipid species in the different study groups. Individual lipids from different lipid classes are shown: (A) lysophosphatidyl choline, (B) sphingomyelin, (C) ethanolamine plasmalogen and (D) cholesteryl ester. Data are shown in mean±SEM for each lipid. Concentration values are expressed in µmol/l [lipid]/mg/dl [HDL protein]. Significance levels are shown as follows: * p<0.05 for Low Hcy group *vs.* placebo group; ** p<0.01 for Low Hcy group *vs.* placebo group; # p<0.05 for Low Hcy group *vs.* High Hcy group; ## p<0.01 for Low Hcy group *vs.* High Hcy group and ### p<0.001 for Low Hcy group *vs.* High Hcy group.

We further examined lipid class-specific lipidomic profiles to confirm the observed changes in the multivariate modeling results and link them with specific lipid molecular changes (Figure 3). LysoPCs were significantly diminished for both fenofibrate groups as compared to placebo group at the study close-out, and this effect was even stronger in High Hcy group. SM levels were significantly elevated in both fenofibrate groups as compared to placebo group at the close-out, and this effect was slightly stronger for the Low Hcy group. Ethanolamine plasmalogen and ChoE levels were elevated in Low Hcy group as compared to the levels in high Hcy group.

### *In silico* reconstitution of HDL particles – the effect of drug induced lipid compositional changes

We then investigated whether the lipid compositional changes induce specific structural differences to spherical HDL particles, especially to the partitioning of different lipid constituents. The change of SM-PC ratio in HDL particles is functionally relevant since SMs are known to effectively solubilize cholesterol and form liquid order phases with cholesterol molecules [24]. Therefore, an increased SM-PC ratio in HDL particles could induce liquid ordered domains in HDL particles, which in turn could affect metabolism and function of HDL.

For the reasons above we carried out, similarly as in our earlier study [21], three simulations to study the effect of SM/PC ratio to the lipid partitioning without changing the phospholipid concentration of particles. We also decreased the lysoPC concentration in one simulation, although removing four lysoPCs from HDL particles does not induce large differences to molecular distribution. The molecular compositions of different molecular dynamics simulation runs are shown in Table 3.

**Table 3:** Molecular composition of different simulation runs.

	SM∶PC∶FCho∶lysoPC∶ChoE∶TG (number of molecules per particle)	apoA-I (number of molecules per particle)	apoA-II( number of molecules per particle)
MD-1	18∶109∶50∶10∶90∶19	2	0
MD-2	25∶102∶50∶6∶90∶19	2	0
MD-3	47∶80∶50∶6∶90∶19	2	0
MD-4	18∶109∶50∶10∶90∶19	2	1

**Figure 4:**
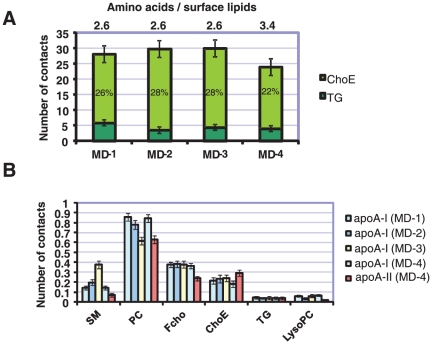
Number of core lipids at the surface and contacts between proteins and different lipids. (A) Number of core lipids at the surface of particles (number of contacts between water and neutral lipid ester beads). When compared to the control simulation (MD-1), the total number of neutral lipids at the surface decreases in simulation MD-4 where the ratio of surface lipids (PC, SM, FCho and lysoPC) and apolipoprotein residues is highest. Bar numbers show the percentage of total neutral lipids at the surface. (B) The number of contacts (per amino acid) between apoA-I or apoA-II and different lipids in simulations. Notably, the affinity of apoA-I towards PC, SM, FCho, and lysoPC is stronger than apoA-IIs. However, the affinity of ApoA-II is stronger towards ChoE compared to apoA-I. Affinities toward TG are same for both apolipoproteins. Errors bars are standard deviations.

We did not detect any marked differences in the localization of TG and ChoE to particle surface monolayer implying that the ratio of SM/PC is not so critical for the solubility of neutral core lipids to surface monolayer in HDL particles (Figure 4). The number of contacts between apoA-I and SM increased as a function of SM concentration. In contrast, the contacts between apoA-I and PC decreased. We did not observe any phase separation in the current molecular dynamics simulations and the preference of cholesterol molecules towards apoA-I did not change. Consequently, the increase of number of contacts between SM and apoA-I with increasing SM-PC ratio is trivial.

### *In silico* reconstitution of HDL particles – the effect of ApoAII

**Figure 5:**
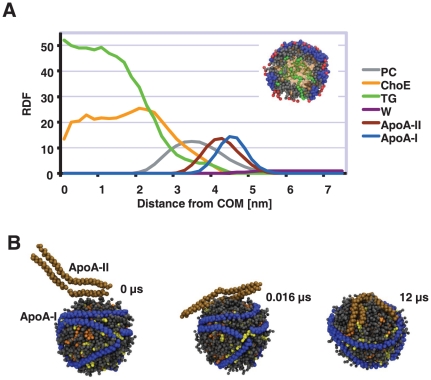
Radial distribution functions of different molecules and snapshots from simualtions. (A) Radial distribution functions for TG, ChoE, PC, apoA-II and apoA-I respect to the center of mass of HDL particle (MD-4). Distributions clearly indicate that apoA-II is more deeply buried into lipid matrix. Inset shows a 2 nm slice from the center of HDL particle. Blue molecule is apoA-I, grey and red spheres are PCs, green ones are TGs, and transparent and orange spheres are ChoEs. (B) Snapshots from simulation MD-4 showing the binding and conformation of apoA-II during simulation MD-4. Top snapshot is from the start of short vacuum simulation. Middle one is from the start of water simulation. Bottom one is from the end of simulation MD-4 showing V-conformation of apoA-II on the surface of HDL particle. PCs and SMs are rendered using grey spheres. Cholesteryl esters and free cholesterol molecules are rendered using orange and yellow spheres, respectively. ApoA-II is rendered using brown spheres and ApoA-I using blue spheres. Only backbone beads are rendered for clarity. Water and TG beads are not shown.

The number of apoA-II molecules differs between individuals in different study groups (Table 1 and Figure 1). The role of apoA-II in the structure of HDL and in HDL as well as in general lipoprotein metabolism is currently poorly understood. We therefore investigated the possible effect of apoA-II on the lipid distribution in HDL particles. Since the amount of apoA-II molecules increased in fenofibrate treated group (especially in Low Hcy individuals), we carried out a simulation with HDL particles composed of two apoA-Is and one apoA-II (lipid composition was the same as in the MD-1 model shown in Table 3). Initially, we placed apoA-II above HDL particle after which we carried out a short vacuum simulation to get apoA-II attached to the surface of HDL particle (Figure 5). Afterwards, we carried out apoA-II HDL particle simulation in water. Snapshot from the end of simulations at 12 µs shows that apoA-II adopted a V-shape-like conformation on the surface of HDL particles and the double belt structure is stable during the whole simulation. In addition, apoA-II did not aggregate with apoA-Is, although the timescales of our simulations is not enough to give reliable estimates of the possible interactions between these proteins.

Next we calculated the number of TGs and ChoEs at the particle surface. Total number of neutral lipids decreased since the binding of apoA-II excluded some of ChoEs from the surface monolayer (Figure 4). This observation is relevant because the binding of apoA-II increases the lipid packing at the surface inducing the exclusion of neutral lipids from the surface monolayer of HDL since neutral lipids are more hydrophobic than phospholipid and cholesterol molecules. Neutral lipids are known to phase separate to phospholipid-air interface in Langmuir monolayer studies with increasing surface pressure [25]. Thus, the increased content of apoA-II in HDL particles increases the surface pressure of amphiphilic monolayer surrounding the hydrophobic core. We hypothesize that the modulation of surface pressure of HDL particles by different apolipoproteins could inhibit the binding of different proteins and enzymes to the HDL surface. For example, it is well known that the addition of human apoA-II to the HDL particle solution excludes human apoA-Is from HDL particles [26], [27]. Consequently, the apoA-II induced remodeling of HDL particles would have marked effects to the metabolism of HDL particles.

We analyzed the lipid affinities of apoA-I and apoA-II by calculating the number of contacts between different lipids and apoA-I/apoA-II. Number of contacts was divided by number of amino acids in apoA-I or apoA-II to produce the lipids affinities per amino acid since the number of amino acids in apoA-II is lower compared to apoA-I. Results indicate that apoA-II has lower affinity towards FCho, PC and SM molecules, but slightly higher affinity towards ChoE as compared to apoA-I. This difference could be partly explained by the double belt structure of apoA-II which decreases the amount of protein surface that can interact with surface lipids. However, at the same time the surface area that is exposed to the core lipids is higher when compared to two individual monomers.

We also compared the binding depth of apoA-I and apoA-II by calculating the radial distribution functions (RDFs) for different molecules with respect to center of mass of HDL particles. RDF profiles show that apoA-II is located deeper in the lipid matrix as compared to apoA-Is, likely reflecting the more hydrophobic nature of apoA-II (Figure 5). This finding suggests and agrees well with previous studies that indicate that the dissociation constant (Kd) of apoA-II is lower than Kd of apoA-I [28].

## Discussion

Herein we explored for the first time the HDL molecular lipid signatures of patients treated with fenofibrate as well as identified the specific lipid species characteristic of high and low Hcy response following the drug treatment. Lipidomic analysis revealed that lysoPCs are diminished following the fenofibrate treatment and that this effect is stronger in patients with high Hcy levels at study close-out. Sphingomyelins (SMs) were upregulated due to fenofibrate treatment. However, according to our molecular dynamics simulations, the increase of SM-PC ratio did not induce marked differences to the distribution of lipids and structure of HDL particles and the solubility of neutral core lipids to surface monolayer did not change with increasing SM-PC ratio. The solubility of neutral lipids to particle surface is functionally important as the amount and type (TG or ChoE) of neutral lipids at the surface could modulate the rate of hydrolysis via the action of hepatic lipase or transfer of neutral lipids that could play an important role in HDL metabolism [29], [30], [31]. The higher the TG content in HDL particle, the better the substrate properties for phospholipid transfer protein (PLTP), which in turn leads to generation of large fused HDL as well as preβ-HDL particles both of which are good acceptors of cholesterol from macrophage-foam cells [32], [33]. In addition, cholesterol molecules seem to have similar affinity towards apoA-I regardless the increase of SM-PC ratio. No phase separation was registered in simulations which is little surprising as it is known that SM and cholesterol are able to form liquid ordered domains [24]. However, HDL particles have high curvature that could play a role in the partitioning behavior of lipids. Also the known limitations of coarse-grained force field could induce unnatural phase behavior of lipids.

ApoA-I is a known cofactor for lecithin-cholesterol acyltransferase (LCAT). Interestingly, Jones and colleagues have suggested that the molecular components (PC and cholesterol molecules) required for ChoE synthesis, must be located near the LCAT activator domain of apoA-I [34], [35]. Furthermore, previous studies indicate that the activity of LCAT decreases when the concentration of SM increases in HDL particles [36], [37] because LCAT is not able to hydrolyze SM molecules to produce free fatty acids. Consequently, the maturation rate of SM-rich HDL particles could be slower than SM-poor HDL particles. This would also affect HDL particle size by a shift towards smaller less protective particles.

ApoA-II was elevated significantly in the fenofibrate-treated individuals as compared to the placebo group. As a result of PPARα-induced apoA-II gene transcriptionhepatic apoA-II production and secretion are elevated [38], but the relevance of apoA-II with respect to atherosclerosis is debated. Our simulation results imply that the apolipoprotein content of HDL particles can modulate the molecular packing at the particle surface, thus affecting the number of neutral lipids at the surface lipid monolayer. ApoA-II also expressed lower affinity towards SM, PC and cholesterol molecules and higher towards ChoE as compared to apoA-I. This may arise from the double-belt like conformation of homodimer apoA-II that reduces the protein area that can be in contact with surface lipids.

Based on our simulation results and earlier experiments we propose that one possible role of apoA-II in HDL metabolism is to modulate the molecular composition of surface monolayer of HDL particles by excluding other apolipoproteins or neutral lipids from the surface monolayer as it possesses higher lipid affinity compared to other apolipoproteins. For example, apoA-IIs can exclude apoA-Is from HDL particles because apoA-II possesses lower dissociation constant [27], [28], [39], [40], [41] that is almost comparable to apoB-100, which is known to be the sole non-exchangeable apolipoprotein in circulation. Simulation data agree with this view since apoA-II was shown to be more deeply buried in HDL particles than apoA-I. Interestingly, apoA-II induced exclusion of apoA-I from HDL could generate more lipid-poor apoA-I molecules (i.e., preβ-HDL) [42] that can actively participate in the early steps of the reverse cholesterol transport. Our data suggests that increase of apoA-II levels by fenofibrates explains, at least partly, the lack of elevation of apoA-I in the circulation.

The mechanism by which fenofibrate increases Hcy levels is poorly understood in humans. Fenofibrate is able to raise HDL-C levels but to a variable degree of response [6], [43], [44]. The greater the increase in Hcy levels the smaller is the increase in the HDL-C levels in fenofibrate treated patients [11]. Identification and understanding of the molecular factors behind the increase of Hcy is clinically important since this may help identify the patients who may benefit from the fenofibrate treatment. We observed that several ether lipids including ethanolamine plasmalogens were elevated in Low Hcy group as compared to High Hcy group due to fenofibrate treatment. Reportedly HDL contains more ethanolamine plasmalogens species compared to other lipoproteins [12]. One of the functions of these plasmalogens is to act as antioxidants and thus prevent the oxidation of cholesterol and phospholipids [45]. Our data therefore suggests that the HDL particles of patients who respond to fenofibrate therapy with elevated Hcy have lower antioxidant capacity. Further studies are needed to address this hypothesis.

As a potential limitation of our study, one has to keep in mind that our simulation model may not represent the real structure of HDL, since the physiological lipid and apolipoprotein compositions of HDL particles are very heterogeneous. However, the *in silico* approach is currently the only available strategy to assess the HDL structure as dependent on lipid molecular changes. Lipid spatial distributions and related trends are to be verified when such experimental information can become available. Furthermore, our findings are clinically relevant also due the unique design of the present fenofibrate-interevention study which allows us to investigate the effect of fenofibrate, placebo and the concerted action of fenofibrate and Hcy.

In conclusion, our study shows that fenofibrate therapy leads to complex compositional changes of HDL particles and that the drug responses are different in patients with elevated Hcy as compared to patients with normal or low Hcy levels following the therapy. The *in silico* studies suggest that the observed compositional changes lead to specific HDL structural changes, with the major effect being linked to the elevated apoA-II level that may also explain at least partly the neutral effect of fenofibrate on apoA-I. The complexity of the fenofibrate response regarding HDL, with some changes pro- and some anti-atherogenic, may explain the heterogeneity of treatment benefits of fibrates, the largest benefits of cardiovascular outcomes being related to the magnitude of TG lowering reported in a recent meta-analyses of fibrate trials [46]. Finally, the present study suggests that more detailed molecular characterization of HDL may provide surrogates for predictors of drug response and thus help identify the patients who might benefit from fenofibrate treatment.

## Materials and Methods

### Study subjects

The FIELD study is a multinational study started in 1998 in Australia, New Zealand and Finland [6]. In the FIELD Helsinki center 270 type 2 diabetic patients were recruited. Of these patients, after exclusion of statin and estrogen users, we selected 33 subjects in the fenofibrate group according to quartile levels of Hcy at 5th year: 17 subjects were in the lowest quartile (Low Hcy) and 16 subjects were in the highest quartile (High Hcy). In addition, 14 subjects allocated to placebo were matched according to Hcy levels at 5^th^ year to Low Hcy group. All the other details of the study subjects are reported elsewhere [47]. Each study subject gave written informed consent before participating in the study. All samples were collected in accordance with the Helsinki Declaration and the Ethics Committee of the Helsinki University Central Hospital approved the study design.

### Biochemical analyses

The baseline serum and plasma samples were collected during the placebo run in period of the FIELD study before any fenofibrate intervention. Serum total cholesterol, triglycerides, HDL-C, LDL-C, apo B, apoA-I and apoA-II were analyzed as described in detail [21]. PLTP activity was measured with a radiometric assay [48]. Plasma homocysteine was determined by a fluorescence polarization immunoassay (Abbott laboratories, Abbott Park, IL, USA) and PON-1 mass using ELISA method (Uscn Life Science Inc., Wuhan, China).

To analyze size distribution of HDL particles, HDL were isolated by ultracentrifugation from 0.5 ml plasma or serum at baseline and at close out and the run in native gradient PAGE [49]. The mean HDL particle size was calculated by multiplying the mean size of each HDL subclass by its relative area under the densitometric scan [50]. The quantification of plasma preβ-HDL levels was performed by crossed immunoelectrophoresis [51].

### Statistical analysis of primary clinical data

The statistical analysis was performed using SPSS 17.0 for Windows (SPSS, Chicago, IL, USA). Data are expressed as mean ± standard deviation Paired sample t test, ANOVA analysis of variance and Kruskall Wallis tests were used for intra-group and between groups comparisons. A *p* value of <0.05 was considered significant.

### Lipidomic analysis

An internal standard mixture 1 was added to each sample of total HDL fraction. The standard mixture contained lysoPC(17∶0), Cer(d18∶1/17∶0), PC(17∶0/17∶0), PE(17∶0/17∶0) and TG(17∶0/17∶0/17∶0) at a concentration level of 0.5–1 µg/sample. Lipids were extracted with chloroform/methanol (2∶1, v/v, 100 µl) solvent. After vortexing (two min) and standing (one hour), the samples were centrifuged at 10000 rpm for 3 min. The lower lipid extract (60 µl ) was added with 10 µl of internal standard mixture 2 containing three labeled lipids: PC (16∶1/0∶0-D_3_), PC(16∶1/16∶1-D_6_) and TG(16∶0/16∶0/16∶0-^13^C3).The samples order was randomised before analysis by Waters Q-Tof Premier mass spectrometer combined with an Acquity Ultra Performance LC™ (UPLC). The column (at 50°C) was an Acquity UPLC™ BEH C18 2.1×100 mm with 1.7 µm particles. The solvent system included (A) Ultrapure water (1% 1 M NH_4_Ac, 0.1% HCOOH) and (B) LC/MS grade acetonitrile/isopropanol (1∶1, 1% 1 M NH_4_Ac, 0.1% HCOOH). The gradient started from 65% A/35% B, reached 80% B in 2 min, 100% B in 7 min, and remained there for 7 min. The flow rate was maintained at 0.400 ml/min and the injected amount was 2.0 µl (Acquity Sample Organizer, at 10°C). Reserpine was used as the lock spray reference compound. The lipid profiling was carried out using ESI in positive mode and the data was collected at a mass range of m/z 300–1200 with scan duration of 0.2 sec.

The data processing using MZmine2 software [52] included alignment of peaks, peak integration, normalization, and peak identification. Lipids were identified using an internal spectral library. The data was normalized using one or more internal standards representatives of each class of lipid present in the samples: the intensity of each identified lipid was normalized by dividing it with the intensity of its corresponding standard and multiplying it by the concentration of the standard. All monoacyl lipids except cholesterol esters, such as monoacylglycerols and monoacylglycerophospholipids, were normalized with PC(17∶0/0∶0), all diacyl lipids except ethanolamine phospholipids were normalized with PC(17∶0/17∶0), all ceramides with Cer(d18∶1/17∶0), all diacyl ethanolamine phospholipids with PE(17∶0/17∶0), and TG and cholesterol esters were normalized with TG(17∶0/17∶0/17∶0). Other (unidentified) molecular species were normalized with PC(17∶0/0∶0) for retention time <300 s, PC(17∶0/17∶0) for retention time between 300 s and 410 s, and TG(17∶0/17∶0/17∶0) for higher retention times.

For further identification of unknown lipids, the sample was analysed with UPLC instrument coupled to chip-based nanoelectrospray (TriVersa Nanomate, Advion Biosciences, Ithaca, NY) and LTQ-Orbitrap mass spectrometer (Thermo Fischer Scientific, San Jose, CA). Fractions (approximately 4 s each) were collected from UPLC run using TriVersa Nanomate and the fractions containing unidentified lipids were infused to a LTQ-Orbitrap mass spectrometer by a TriVersa Nanomate in positive and negative ionisation mode. Identifications were based on the exact mass and MS^n^ spectra. The instrument was calibrated externally according to the instructions of manufacturer. MS^2^ and MS^3^ were acquired using either low resolution (LTQ) or high resolution up to target mass resolution R = 60,000 at m/z 400. The normalized collision energies of 30–40% were applied in MS^n^ experiments.

### Multivariate multi-way models

To analyze the effects of covariates on lipidomic profiles we used a recently published methodology [22], [23] developed to extend ANOVA-type multivariate multi-way analysis to high-dimensional, small sample-size setups. The method is particularly suitable for analysing populations of high-dimensional lipidomics (or other continuous-valued ‘omics’) profiles, where each sample (individual patient) is associated to multiple covariates (such as disease and treatment). The method assumes that there are strongly correlated, similarly behaving clusters of lipids in the data, and models the effects of the covariates and their interactions for each cluster. The method is based on a unified Bayesian model, in which both the clustering and of the multiple covariate effects are estimated simultaneously.

There are several advantages in this modeling approach. Modeling the effects of covariates on clusters of variables is an excellent strategy against multiple testing problems. Secondly, Bayesian methods take uncertainties in the data properly into account which helps avoid over-fitting. In addition, the uncertainty due to the small number of samples becomes evaluated and reported properly. Finally, in contrast to resorting to *a priori* defined clusters, such as gene sets in gene expression analyses, the method finds the clusters as a part of the analysis, making it possible to find novel clusters of lipids which both behave similarly and are informative of the covariate effects.

The method was originally published for a standard multi-way design that can be described as a linear model consisting of main effects and interaction effects. In our dataset there are three groups of individuals: low Hcy – fenofibrate, high Hcy – fenofibrate, and low Hcy – placebo. The lipidomics profiles have been measured before and after treatment. The experimental design is slightly more complicated than the standard one assumed in [22] in two respects: (1) There are no observations in the group high-Hcy-placebo. The reason is that this group is biologically impossible: placebo does not raise Hcy levels of any patients considerably. (2) The measurements were taken from each patient before and after the treatment, requiring a repeated measures treatment (pairwise comparisons).

We extended the model to incorporate the two new assumptions, still allowing us to model the dataset within a single unified model. The multi-way design including repeated-measures terms was implemented as follows:

The multivariate multi-way model is a hierarchical Bayesian model
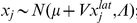
and the effects of covariates operate on a latent factor space for clusters of lipids, as explained in [22].

Before treatment:

Normal Hcy placebo: 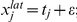


Normal Hcy fenofibrate: 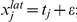


High Hcy fenofibrate: 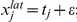


After treatment:

Normal Hcy placebo: 
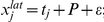


Normal Hcy fenofibrate: 
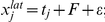


High Hcy fenofibrate: 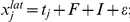


where 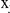 is a vector of concentrations of lipids for patient 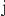, 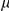 is the grand mean (average of lipid levels before treatment), 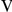 is the projection matrix, 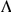 is a diagonal matrix of residual variances, 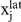 is a latent factor for patient 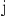, and 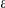 is a noise term. The 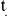 is the patient-specific effect for patient 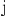, being the same before and after treatment. The 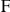 is the fenofibrate effect, 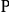 is the placebo effect, 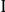 is the interaction of fenofibrate and Hcy level.

In the standard formulation of repeated measures designs in ANOVA-type linear models, a “patient-specific effect” term is included into the model; this term is specific to the individual. The ANOVA test for studying before/after-type of changes is stronger when individual variation in samples has been modeled out with such a term. Although repeated measures are a standard practice in classical ANOVA studies, we are not aware of any work where individual effects would have been included in Bayesian models where the modeling is done in the latent variable space. We implemented such a design and checked that it works well on data with known effects.

The model parameters were estimated by Gibbs sampling discarding 2000 burn-in samples and collecting 2000 Gibbs samples. The optimal number of clusters was in the range of 20–25, determined by predictive likelihood [22]. We used 25 clusters, resulting in 22 non-empty clusters.

### Construction of simulation systems and simulation parameters

In order to study the effect of SM concentration to HDL properties we used the end structure of our normal-HDL (we refer to this MD-1 here) model from the previous simulation study [21]. We randomly replaced PCs molecules by SM molecules. In the first simulation we increased the number of SMs by 30% and reduced the number of PCs by 6% and lysoPCs by 30%. We refer to this simulation by MD-2. In addition to this, we did additional simulation with 260% increase of SM content and 27% decrease of PCs content to find out if more pronounced SM content increase is able to change the distribution and phase behavior of lipids in HDL particle (MD-3). The exact particle numbers in simulations are shown in Table 3.

For apoA-II simulation we also used the MD-1 as a starting system. To this structure we directly incorporated apoA-II which was coarse grained from the X-ray structure of aggregated apoA-II [53]. We generated dimeric apoA-II where disulphide bond is present between Cys-6 residues of each monomer. The secondary structure of Apo-II was almost completely enforced to be alpha-helical. However, coil regions were generated to the middle of structure based on the information derived from X-ray structure where a structural kink was present near residues 34–36. The flexibility of alpha-helical segments arises from the three-body angle and four-body dihedral potentials of backbone beads [54]. Dimeric apoA-II was placed near the surface of HDL particle so that the hydrophobic side was pointing towards lipids and the distance between lipid surface and N-terminal part of apoA-II was approximately 0.5 nm. After the placement of apoA-II short vacuum simulation were carried out to bind apoA-II to lipid matrix. Afterwards system was solvated. Previously, we have used similar approach to study lipid composition effects to spatial distribution of lipids [21], [55]. We refer to the apoA-II simulation by MD-4.

All systems were first energy minimized by the steepest descent algorithm. MD-2 and MD-3 simulations were simulated up to 2.5 µs, which corresponds to 10 µs and more as effective time since the MARTINI model speeds up the dynamics by an approximate factor or four or more [54]. Last 5 µs was used in the analysis. Production simulations for apoA-II simulation MD-4 (non-fused) lasted for 3 µs, which corresponds to 12 µs and more as effective time. System sizes were approximately 26000 water beads and 5000 lipid and protein beads in total. The box size in each non-fused simulation was 15×15×15 nm.

Simulations were performed by the GROMACS simulation package (v. 4.5.1) [56] and the standard Martini lipid force field was used for PC (PC(16∶0/18∶1)), lysoPC (PC(16∶0/0∶0)), FCho and SM (SM(d18∶1/16∶0)) molecules [57]. Protein part was modeled using the protein extension of the Martini description [54], TG (TG(18∶1/18∶1/18∶1)) and ChoE (ChoE(18∶1)) force fields were constructed based on the philosophy of Martini force field, the exact parameters and validation are available elsewhere [55]. In all simulations temperature was set to 310 K and pressure to 1 bar. Berendsen temperature and pressure coupling algorithms [58] were utilized with coupling constants of 0.4 ps and 2.0 ps, respectively. All lipid classes, protein residues and water were separately coupled to heat bath. Electrostatic and Lennard-Jones interactions were calculated using the shift type potentials with cut-off lengths of 1.2 nm and the potentials were shifted to zero starting at 0.0 and 0.9 nm, respectively. Time step was set to 0.025 ps. Systems were treated equilibrated when there was no drift in number of contacts between apoA-I or apoA-II and different lipids. In addition, there was no drift in the number of contacts between ester bead regions of neutral lipids and water molecules. We have to stress that conformation of proteins is not in equilibrium but partitioning of helixes is broadened. Gromacs analysis programs g_mindist and g_rdf were used in the analysis. VMD was used to produce the figures from trajectory snapshots [59].
